# Epilepsy surgery for low-grade epilepsy-associated neuroepithelial tumor of temporal lobe: a single-institution experience of 61 patients

**DOI:** 10.1007/s10072-021-05703-3

**Published:** 2021-11-24

**Authors:** Zhe Zheng, Hongjie Jiang, Hemmings Wu, Yao Ding, Shuang Wang, Wenjie Ming, Junming Zhu

**Affiliations:** 1 Epilepsy Center, The Second Affiliated Hospital, Zhejiang University School of Medicine, No. 88 Jiefang Road, Shangchen District, Hangzhou, 310009 China; 2 Department of Neurosurgery The Second Affiliated Hospital, Zhejiang University School of Medicine, No. 88 Jiefang Road, Shangchen District, Hangzhou, 310009 China; 3 Department of Neurology, The Second Affiliated Hospital, Zhejiang University School of Medicine, No. 88 Jiefang Road, Shangchen District, Hangzhou, 310009 China

**Keywords:** Low-grade epilepsy-associated neuroepithelial tumor, Temporal lobe, Surgery, Seizure, Electrocorticogram, Extended resection

## Abstract

**Background:**

Low-grade epilepsy-associated neuroepithelial tumor (LEAT) is highly responsive to surgery in general. The appropriate surgical strategy remains controversial in temporal LEAT. The aim of this study is to analyze the surgical seizure outcome of temporal LEAT, focusing on the aspects of surgical strategy.

**Methods:**

Sixty-one patients from a single epilepsy center with temporal LEAT underwent surgery. The surgical strategy was according to the multidisciplinary presurgical evaluation. Electrocorticogram (ECoG)-assisted resection was utilized. Surgical extent including lesionectomy and extended resection was described in detail. Seizure outcome was classified as satisfactory (Engel class I) and unsatisfactory (Engel classes II–IV).

**Results:**

After a median follow-up of 36.0 (30.0) months, 83.6% of patients achieved satisfactory outcome, including 72.1% with Engel class Ia. There was 39.3% (24/61) of patients with antiepileptic drug (AED) withdrawal. Use of ECoG (χ2 = 0.000, *P* > 0.1), preresection spike (χ2 = 0.000, *P* = 0.763), or spike residue (*P* = 0.545) was not correlated with the seizure outcome. For lateral temporal LEAT, outcome from lesionectomy was comparable to extended resection (χ2 = 0.499, *P* > 0.1). For mesial temporal LEAT, 94.7% (18/19) of patients who underwent additional hippocampectomy were satisfactory, whereas only 25% (1/4) of patients who underwent lesionectomy were satisfactory (*P* = 0.009).

**Conclusion:**

Surgical treatment was highly effective for temporal LEAT. ECoG may not influence the seizure outcome. For lateral temporal LEAT, lesionectomy with or without cortectomy was sufficient in most patients. For mesial temporal LEAT, extended resection was recommended.

## Introduction

Low-grade epilepsy-associated neuroepithelial tumors (LEAT) are the second-largest histopathological category in epilepsy surgery, just after hippocampal sclerosis [1]. LEAT comprises glioneuronal tumor and low-grade glial tumor [2, 3]. Glioneuronal tumor such as ganglioglioma (GG) and dysembryoplastic neuroepithelial tumor (DNET) are the most common types [4–6]. The frequently encountered histotypes of low-grade glial tumor include pleomorphic xanthoastrocytoma (PXA), pilocytic astrocytoma (PA), oligodendroglioma, and diffuse astrocytoma [2, 7]. LEAT is generally slow-growing tumor, often arises in children and young adults. LEAT is located mostly in the temporal lobe and sometimes coexist with focal cortical dysplasia (FCD) [3, 8]. Hippocampal sclerosis could also occur in temporal LEAT [9], which increases the complexity and treatment difficulty of temporal LEAT.

Long-term satisfactory seizure outcome could be achieved by surgery in most patients with LEAT [1, 3, 6, 7, 10]. However, consensus on surgical strategy has not yet been established. For extratemporal LEAT, lesionectomy seems to be the preferred treatment option with favorable outcome [4, 11, 12]. For temporal LEAT, surgical strategy is more complicated. First, the influence of electrocorticogram (ECoG) on surgical outcome remains controversial [13–15]. Second, some studies suggested extended resection guided by presurgical evaluation and/or ECoG [13, 16–18], while other studies indicated no additional benefits obtained from extended resection [3, 14, 15, 19, 20]. Third, the location of LEAT in the lateral or mesial part may result in completely different surgical approach. In addition, considering the balance between seizure reduction and risk of memory decline, it is more difficult to determine the optimal extent of resection when the mesial temporal structures are involved [9, 21].

The aim of this study is to analyze the surgical outcome in terms of seizure control of temporal LEAT from a single epilepsy center, focusing on the aspects of ECoG and surgical extent.

## Materials and methods

### Patients

Between January 2013 and January 2020, 89 patients with LEAT underwent surgery in the epilepsy center of the Zhejiang University School of Medicine Second Affiliated Hospital. Inclusion criteria were as follows: tumor located solely in the temporal lobe without extending into other lobes, WHO grade I or II verified by pathological examination, and follow-up of at least 12 months. Patients with previous brain surgery or MRI-verified findings of other brain lesions such as cavernous malformation were excluded. Three patients lost during follow-up and 25 patients with extratemporal lobe LEAT were excluded. Sixty-one patients were included in this retrospective study. This study was approved by the hospital ethics committee.

### Presurgical evaluation

In all patients, seizure was the first and only manifestation. Detailed epilepsy history and manifestation were documented. Data used for the analysis included the following clinical and demographic parameters: age of seizure onset, duration of epilepsy, age of surgery, seizure type, seizure frequency, and antiepileptic drugs (AEDs). Drug resistance was defined as failure to long-term (at least 1 year) adequate trials of two or more first-line AEDs. As for seizure frequency, patients with only 1 or 2 seizure attacks were categorized as sporadic group. For patients with sporadic seizures or annual seizures, 24-h video electroencephalography (VEEG) (580-G2CGS S32, Biologic or EEG-1200C, Nihon Kohden) was advised. Ictal capture was considered usually in patients with more frequent seizures or drug-resistant epilepsy. Invasive stereoelectroencephalography (SEEG) was performed in 1 patient to determine whether the hippocampus could be reserved with left amygdala small lesion.

All patients underwent 1.5-T (Siemens, Germany) or 3-T MRI (GE, Germany) scans. Preoperative MRI images were all available in the hospital database except for 2 patients. MRI parameters included tumor laterality, exact location and anatomic structures involvement, and tumor volume. There was no coexistence of focal cortical dysplasia or hippocampal sclerosis in the presurgical MRI. Mesial temporal LEAT was determined if the tumor involved hippocampus, parahippocampal gyrus, amygdala, or uncus. Tumor volume was calculated by the formula of 4/3 × π × A × B × C, where *A*, *B*, and *C* are the mediolateral, dorsoventral, and anteroposterior dimensions, respectively. Preliminary surgical strategy was then made by a multidisciplinary team.

### Surgical strategy

All surgeries were performed by one of the coauthors (JM Zhu). For patients with lateral temporal LEAT, resection sparing the mesial temporal structures was routinely performed. Two patients with frequent spikes in the hippocampus and 1 patient with tumor in the fusiform gyrus underwent additional resection of mesial structures. For patients with mesial temporal LEAT, amygdalohippocampectomy was the first choice. Anterior temporal lobectomy (ATL) was usually performed for better exposure in the early stage of this study. Then, the surgical strategy was transformed to SAH through superior temporal sulcus. Resection sparing the hippocampus was performed in 4 patients to prevent memory decline or due to technical challenges.

ECoG (NicoletOne, USA) use was not randomized, which was determined by the appointment of epileptic electrophysiologist (WJ Ming) and economic burden of patients. In most patients who underwent ECoG, the surgery was assisted by the ECoG. When the tumor was located in the language area (3 patients) or when selective amygdalohippocampectomy (SAH) was performed, the extent of resection was just according to the presurgical evaluation. Propofol-based general anesthesia was performed; after 15-min stop of propofol, 4-contact or 8-contact strip (Sinovation, China) was placed on the surface of superior temporal gyrus, middle temporal gyrus, inferior temporal gyrus, basal temporal gyrus, and tumor area. When single spike, polyspikes, or rhythmic spikes were found, extended resection was performed. Lesionectomy was defined as surrounding brain tissue removal less than 0.5 cm.

### Follow-up

Two patients with glial tumor underwent postoperative radiotherapy. AED withdrawal was advised when the patient reached and remained seizure-free for at least 1 year.

Follow-up information regarding seizure outcome, AEDs, and surgical complication was obtained from regular yearly outpatient visit and telephone interviews. Seizure outcome was assessed according to the Engel classification [22]. Engel class I include the following: Ia, complete seizure-free; Ib, non-disabling simple partial seizures only; Ic, some disabling seizures after surgery, but free of disabling seizures for at least 2 years; and Id, generalized convulsions with AED withdrawal only. Patients with Engel class I outcome were assigned to satisfactory group, and those with Engel classes II–IV outcome to unsatisfactory group. MRI scans were advised at 6-month postoperation, and then yearly after. VEEG was advised 6 months postoperatively.

### Statistical analysis

Continuous data were presented as mean ± SD (standard deviation) or median (interquartile range) based on the normality and homogeneity of variance. Differences between two groups were analyzed using Student *t* test or non-parametric test according to the normality and homogeneity of variance. Differences between groups were analyzed using chi-square test, continuity correction, or Fisher’s exact test. All statistical analyses were performed using SPSS Version 24 (SPSS Inc, Chicago, IL, USA). Statistical significance was indicated at *P* < 0.05. Univariate factors with *P* < 0.05 were included in the multivariate logistic analysis.

## Outcome

There were 31 (50.8%) male and 43 (70.5%) adults. The median age at surgery was 24.0 (14.0), and the median age of seizure onset was 20.0 (17.5). The median duration of epilepsy was 2.0 (5.0) years. Twenty-eight (45.9%) patients were drug resistant, and secondary generalized colonic tonic seizure (GCTS) occurred in 67.2% of patients.

### Presurgical VEEG and Imaging

Fifty (85.2%) of 61 patients underwent VEEG. Interictal VEEG was localized to the affected temporal lobe in 30 out of 45 (66.7%) localized patients and non-localized in 7 (13.5%) patients. Ictal VEEG was available in 29 patients, showing localization to the affected temporal lobe in 18 (62.1%) patients, and non-concordant localization in 1 patient.

The tumor was located in the left hemisphere in 50.8% (31/61) of patients, and in the mesial site in 37.7% (23/61) of patients. The median tumor volume was 16.9 (23.8) cm^3^. PET data indicated hypometabolism in the tumor area in 82.4% (14/17) of patients. Hypermetabolism and normal metabolism were showed in the area of tumor in 2 patients and 1 patient, respectively. Besides, hypometabolism of distant area was showed in 2 patients.

### Surgical outcome

**Fig.1:**
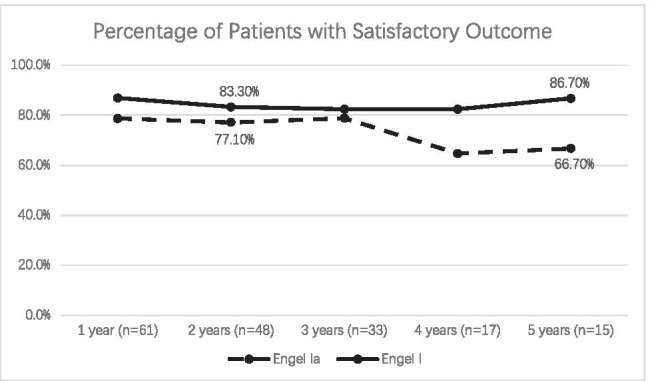
Longitudinal satisfactory (Engel class I vs Ia) seizure outcome of 61 patients with temporal LEAT

The median follow-up period was 36.0 (30.0) months. 83.6% (51/61) of patients were Engel class I, including 44 (72.1%) patients with Engel class Ia. Four patients were Engel class II, 2 patients were Engel class III and 4 patients were Engel class IV. The proportion of Engel class I was 83.3% at the second postoperative year and 86.7% at the fifth year (Fig. 1). The most common pathology was ganglioglioma, which constitute 57.4% (35/61) of all cases, followed by PXA (11), DNET (5), PA (4), oligodendroglioma (3), oligoastrocytoma (3), and undefined tumor (2). There were 41 patients with glioneuronal tumor and 45 patients with WHO grade I tumor. Total tumor removal was achieved in 91.8% (56/61) of patients. Associated FCD and hippocampal sclerosis were pathologically verified in 3 and 1 patient, respectively. Hippocampal gliosis was reported in 6 patients. No tumor progression was found during the follow-up. The only predictor for seizure outcome was age at surgery (*Z* =  − 2.417, *P* = 0.016, OR = 0.942, 95% CI = 0.892 ~ 0.993).

There was no morality or severe neurological deficits. Only 1 patient reported mild speech disturbance.

### ECoG and extent of resection

ECoG was performed in 83.6% (51/60) of patients. No difference was found between the patients with ECoG and those without ECoG in the clinical characteristics (Table 1) and seizure outcome (χ^2^ = 0.000, *P* > 0.1) (Table 2). Preresective spike (χ^2^ = 0.091, *P* = 0.763) or spike residue (*P* = 0.545) was not correlated with the seizure outcome (Table 2).

**Table 1:** Comparison between two groups of 60 patients with or without ECoG

	ECoG	No ECoG	Statistics	*P*
Gender			χ^2^ = 0.378	0.539
Male	25 (80.6%)	6 (19.4%)		
Female	26 (89.7%)	3 (10.3%)		
Age			χ^2^ = 0.000	> 0.1
Children	15 (83.3%)	3 (16.7%)		
Adults	36 (85.7%)	6 (14.3%)		
GCTS			χ^2^ = 0.000	> 0.1
Yes	35 (85.4%)	6 (14.6%)		
No	16 (84.2%)	3 (15.8%)		
Drug resistance			χ^2^ = 1.269	0.260
Yes	25 (92.6%)	2 (7.4%)		
No	26 (78.8%)	7 (21.2%)		
Seizure frequency			χ^2^ = 0.338	0.953
Sporadic	7 (87.5%)	1 (12.5%)		
Annual	8 (88.9%)	1 (11.1%)		
Monthly	28 (84.8%)	5 (15.2%)		
Daily	8 (80.0%)	2 (20.0%)		
Age at surgery (year)	24.5 (14.5)	22.0 (12.3)	Z = -0.797	0.425
Age at seizure onset (year)	19.0 (18.3)	21.0 (20.0)	Z = -0.383	0.701
Duration of epilepsy (year)	2.0 (5.0)	1.0 (6.4)	Z = -0.436	0.663
Tumor laterality			χ^2^ = 0.000	> 0.1
Left	25 (83.3%)	5 (16.7%)		
Right	26 (86.7%)	4 (13.3%)		
Tumor location			χ^2^ = 2.724	0.099
Mesial	16 (72.7%)	6 (27.3%)		
Lateral	35 (92.1%)	3 (7.9%)		
Tumor volume (cm^3^)	17.0 (25.5)	22.8 (72.6)	Z = -0.992	0.321
Tumor remnant			-	0.103
No	49 (87.5%)	7 (12.5%)		
Yes	2 (50.0%)	2 (50.0%)		
Resection extent			χ^2^ = 0.000	> 0.1
Lesionectomy	9 (90.0%)	1 (10.0%)		
Extended resection	42 (84.0%)	8 (16.0%)		
Pathology (1 undefined glioneuronal tumor)			χ^2^ = 0.732	0.392
Ganglioglioma	28 (80.0%)	7 (20.0%)		
Non-ganglioglioma	22 (91.7%)	2 (8.3%)		
WHO grade			χ^2^ = 0.293	0.589
I	37 (82.2%)	8 (17.8%)		
II	13 (92.9%)	1 (7.1%)

**Table 2:** Seizure outcome and ECoG (*n* = 60)

	No. of patients	Engel I	Engel II–IV	Statistics	*P*
ECoG				χ^2^ = 0.000	> 0.1
Yes	51	43 (84.3%)	8 (15.7%)		
No	9	7 (77.8%)	2 (22.2%)		
Spike before resection				χ^2^ = 0.091	0.763
Yes	36	30 (83.3%)	6 (16.7%)		
No	15	13 (86.7%)	2 (13.3%)		
Spike after resection				-	0.545
Yes	9	9 (100.0%)	0 (0.0%)		
No	24	21 (87.5%)	3 (12.5%)		

Three patients with preoperative spike did not undergo ECoG after resection due to presurgical plan of selective resection of temporal mesial structures

The details of tumor location and surgical extent are shown in Table 3. Fifty-one (83.6%) out of 61 patients underwent extended resection, and no difference in terms of seizure outcome was found between lesionectomy and extended resection (*P* = 0.345). In the 38 patients with lateral temporal LEAT, lesionectomy, extended cortectomy, and anterior lateral lobectomy were performed in 8, 18, and 12 patients, respectively. No difference in the seizure outcome was found between these groups (χ^2^ = 0.499, *P* > 0.1). No difference was found in seizure outcome between patients with lateral temporal LEAT involving fusiform gyrus and those without (*P* = 0.295). Among the 23 patients with mesial temporal LEAT, 94.7% (18/19) of patients got satisfactory seizure outcome with hippocampectomy, while only 25.0% (1/4) of patients achieved satisfactory seizure outcome without hippocampectomy (*P* = 0.009) (Table 4).

**Table 3:** Seizure outcome, tumor location, involvement, and type of surgery

Tumor location	Tumor involvement	No. of patients	Type of surgery	Engel I		No. of patients
				Yes	No	
Mesial temporal lobe	H involvement	8	L	0	1	1
			SAH + L	4	0	4
			ATL + AH + L	3	0	3
	H not involvement	15	L	0	1	1
			A + L	1	1	2
			SAH + L	1	0	1
			ATL + AH + L	10	1	11
Lateral temporal lobe	FG involvement	11	L	2	0	2
			EC + L	4	0	4
			ATL + L	4	0	4
			ATL + AH + L	1	0	1
	FG not involvement	27	L	5	1	6
			EC + L	12	2	14
			ATL + L	3	2	5
			ATL + AH + L	2	0	2
Total				52	9	61

*H* hippocampus; *L* lesionectomy; *SAH* selective amygdalohippocampectomy; *ATL* anterior temporal lobectomy; *AH* amygdalohippocampectomy; *A* amygdalectomy; *FG* fusiform gyrus; *EC* extended cortectomy

**Table 4:** Demographic comparison of patients with satisfactory (Engel class I) and unsatisfactory (Engel class II-IV) seizure outcome

	Engel I	Engel II–IV	Statistics	*P*
	*N* (%)	*N* (%)		
Gender			χ^2^ = 0.000	> 0.1
Male	26 (83.9%)	5 (16.1%)		
Female	25 (83.3%)	5 (16.7%)		
Age			χ^2^ = 1.210	0.271
Children	17 (94.4%)	1 (5.6%)		
Adults	34 (79.1%)	9 (20.9%)		
GCTS			χ^2^ = 0.000	> 0.1
Yes	34 (82.9%)	7 (17.1%)		
No	17 (85.0%)	3 (15.0%)		
Drug resistance			χ^2^ = 0.000	> 0.1
Yes	23 (82.1%)	5 (17.9%)		
No	28 (84.8%)	5 (15.2%)		
Seizure frequency			χ^2^ = 0.425	0.935
Sporadic	7 (87.5%)	1 (12.5%)		
Annual	8 (88.9%)	1 (11.1%)		
Monthly	28 (82.4%)	6 (17.6%)		
Daily	8 (80.0%)	2 (20.0%)		
Age at surgery (year)	23.0 (14.0)	28.5 (21.0)	Z = -2.417	0.016
Age at seizure onset (year)	20.0 (16.0)	13.5 (15.3)	Z = -0.761	0.447
Duration of epilepsy (year)	1.5 (4.3)	14.0 (49.8)	Z = -1.134	0.257
Tumor laterality			χ^2^ = 3.190	0.074
Left	29 (93.5%)	2 (6.5%)		
Right	22 (73.3%)	8 (26.7%)		
Tumor location			χ^2^ = 0.000	> 0.1
Mesial	19 (82.6%)	4 (17.4%)		
Lateral	32 (84.2%)	6 (15.8%)		
Tumor volume (cm^3^)	15.9 (18.0)	12.6 (169.6)	χ^2^ = -0.545	0.585
Tumor remnant			-	0.185
No	48 (85.7%)	8 (14.3%)		
Yes	3 (60.0%)	2 (40.0%)		
Resection extent			-	0.345
Lesionectomy	7 (70.0%)	3 (30.0%)		
Extended resection	44 (86.3%)	7 (13.7%)		
Resection extent of lateral temporal lobe			χ^2^ = 0.499	> 0.1
Lesionectomy	7 (87.5%)	1 (12.5%)		
EC	16 (88.9%)	2 (11.1%)		
ATL	10 (83.3%)	2 (16.7%)		
Resection extent of mesial temporal lobe			-	0.009
No hippocampectomy	1 (25.0%)	3 (75.0%)		
Hippocampectomy plus	18 (94.7%)	1 (5.3%)		
Pathology (1 undefined glioneuronal tumor)			χ^2^ = 0.034	0.855
Ganglioglioma	29 (82.9%)	6 (17.1%)		
Non-ganglioglioma	22 (88.0%)	3 (12.0%)		
WHO grade(1 not classified)			χ^2^ = 0.640	0.424
I	36 (80.0%)	9 (20.0%)		
II	14 (93.3%)	1 (6.7%)		

*GCTS* generalized colonic tonic seizure; *EC* extended cortectomy; *ATL* anterior temporal lobectomy

### Postoperative AEDs

Of patients, 39.3% (24/61) was AED withdrawal, and 50.8% (31/61) of patients used only 1 kind of AEDs, while 6 patients used 2 or more ADEs at last follow-up. Twenty-seven patients attempted AED withdrawal, and 3 patients had seizure recurrence, and 1 of them regained seizure control with AEDs.

## Discussion

In this present study of 61 patients with temporal LEAT, after a median follow-up period of 36.0 (30.0) months, 83.6% (51/61) of patients were Engel class I, and 72.1% (44/61) of them achieved Engel class Ia. These results were in line with other similar series, in which 69% to 87% of patients were free of disabling seizures after surgery [6, 21, 23–26]. The proportion of Engel class I was stable, from 83.3% at the second year to 86.7% at the fifth year postoperation. Comparable findings were shown by Phi et al., with rate of seizure free from 86% at the second year to 79% at the fifth year [9]. Surgical treatment was highly effective for temporal LEAT in terms of seizure control.

Total resection of temporal LEAT was reported to be correlated with better seizure outcome [9, 27]. However, data from our study did not support this view. There were only 5 patients with tumor remnant; small number may influence the deduction of meaningful statistical conclusion. The only statistical significant prognostic factor was age at surgery (*P* = 0.016, OR = 0.942), as previous studies also suggested that younger age predicted better outcome [3, 5]. Earlier surgery was recommended due to better seizure outcome and better postoperative cognitive function in children [3, 28].

ECoG was widely applied in epilepsy surgery, but its application was limited in LEAT surgery due to its uncertain advantages. ECoG may prolong the surgical procedure, increase the cost, and may increase the surgical extent and risk. Thus, the impact of ECoG on LEAT surgery was still under debate. In this study, the use of ECoG did not provide additional benefits to seizure outcome. Though ECoG was not randomized, patients with ECoG and those without ECoG were comparable in the clinical characteristics. Some previous studies also indicated that ECoG use did not predict the seizure outcome [4, 14, 15]. However, some authors showed that ECoG-guided extended resection could achieve better seizure outcome [13, 29]. Our series indicated that spike residue did not influence the seizure outcome. Similar conclusion was also reported by Wray et al. [30]. Interestingly, recent study showed that fast ripple residue in ECoG signals was correlated with worse seizure outcome [31]. ECoG was still controversial in temporal LEAT surgery, and randomized studies were warranted to further validate its value.

Some studies indicated that lesionectomy may result in low rate of favorable seizure outcome in temporal tumor–associated epilepsy, while extended resection significantly improved the outcome [13, 29]. However, no difference was found between lesionectomy and extended resection in our series. Uliel-Sibony et al. indicated that extended resection did not provide additional benefits when total resection was achieved [25]. Even when standard temporal lobectomy was performed, there was still around 20% of patients with unsatisfactory seizure outcome [32]. Our data suggested that the more relevant issue is whether the mesial structure is involved, which may influence the extent of resection and seizure outcome.

In our series with mesial temporal LEAT, extended resection including additional hippocampectomy resulted in better seizure outcome (*P* = 0.009). Giulioni et al. [33] studied patients with mesial temporal tumor and refractory epilepsy, and favorable seizure outcome was achieved in 93% of patients with extended resection, and only 42.5% of those with lesionectomy. Lesionectomy may be not enough in mesial temporal LEAT, and additional mesial structure removal was recommended [6, 9, 23, 33]. However, the risk of memory decline due to hippocampus removal should be considered, especially on the dominant side [34]. To obtain better seizure outcome, we performed hippocampectomy in most patients with mesial temporal LEAT. Several epilepsy centers have showed that extended resection sparing the dominant hippocampus without sacrificing seizure outcome was possible when the tumor was located in the amygdala or uncus after detailed presurgical evaluation [9, 35].

In our series of lateral temporal LEAT, 92.1% (35/38) of patients underwent resection sparing the mesial structures, and 85.7% of patients had satisfactory seizure outcome. Although lesionectomy and extended resection resulted in similar seizure outcome (*P* > 0.1), selective bias may exist. Based on that and outcome of ECoG of our epilepsy center, lesionectomy with or without more limited cortectomy other than large-area extended resection will be tried in patients with lateral temporal LEAT in the future. However, whether lesionectomy alone was sufficient for lateral temporal LEAT was not clear yet, and extended resection (0.5 ~ 2 cm) around the tumor was advised by some authors [24, 34]. More detailed individual presurgical evaluation, such as PET, neuropsychological test, and even SEEG, may shed light on this subject [23]. When it comes to the question of management of the mesial structures, there is no standard criterion, and the only common consensus is that hippocampus should be removed if there is hippocampal sclerosis or abnormal signal in presurgical MRI [9, 24, 25].

Of patients, 39.3% (24/61) had AED withdrawal in our study. Another study reported 2-year and 5-year AED withdrawal rates at 42.9% and 72%, respectively [9]. Not long enough, follow-up (median 3 years) in the present study may influence the final proportion of AED withdrawal. AED withdrawal contributed improvement in neurological functions [36]. The best time of AED withdrawal for LEAT remained debatable. Usually AED withdrawal was recommended after at least 1 to 2 years of seizure-free. Early AED withdrawal had significant risk for seizure recurrence [37], but it did not influence the final seizure outcome, and most patients with seizure recurrence after AED withdrawal could regain seizure-free after readministration of AEDs [38]. Higher rate of AEDs withdrawal could be achieved in children compared to adults [1, 5]. Another study showed that patients with LEAT had the highest rate of 5-year AED withdrawal compared to other epilepsy pathologies [1]. Early surgical treatment for LEAT was recommended.

Firstly, this study was retrospective and non-randomized; selection bias may influence the outcome. Secondly, the number of patients was small, especially in some subgroups, which may influence the outcome of analysis. Thirdly, the surgical extent was too heterogeneous, not only according to the presurgical evaluation, but also referred to the ECoG, detailed deduction was hard to perform to obtain powerful conclusion.

## Conclusion

Surgical treatment was highly effective and safe for temporal LEAT. ECoG was not correlated with seizure outcome. For mesial temporal LEAT, extended resection was recommended. For lateral temporal LEAT, lesionectomy with or without cortectomy sparing the mesial structures was appropriate in most patients.
